# Supplementary material for: Saturation mutagenesis of twenty disease-associated regulatory elements at single base-pair resolution
Source: Nat Commun. 2019 Aug 8;10:3583. doi: 10.1038/s41467-019-11526-w (PMC6687891; doi:10.1038/s41467-019-11526-w)
Supplement: Supplementary file 2 — Reporting Summary [file 41467_2019_11526_MOESM2_ESM.pdf]

nature research

Corresponding author(s): Martin Kircher, Jay Shendure, Nadav Ahituv

Last updated by author(s): Jul 3, 2019

Reporting Summary

Nature Research wishes to improve the reproducibility of the work that we publish. This form provides structure for consistency and transparency in reporting. For further information on Nature Research policies, see Authors & Referees and the Editorial Policy Checklist.

Statistics

For all statistical analyses, confirm that the following items are present in the figure legend, table legend, main text, or Methods section.

n/a	Confirmed	
☐	☒	The exact sample size (*n*) for each experimental group/condition, given as a discrete number and unit of measurement
☐	☒	A statement on whether measurements were taken from distinct samples or whether the same sample was measured repeatedly
☐	☒	The statistical test(s) used AND whether they are one- or two-sided<br>*Only common tests should be described solely by name; describe more complex techniques in the Methods section.*
☐	☒	A description of all covariates tested
☐	☒	A description of any assumptions or corrections, such as tests of normality and adjustment for multiple comparisons
☐	☒	A full description of the statistical parameters including central tendency (e.g. means) or other basic estimates (e.g. regression coefficient) AND variation (e.g. standard deviation) or associated estimates of uncertainty (e.g. confidence intervals)
☐	☒	For null hypothesis testing, the test statistic (e.g. *F*, *t*, *r*) with confidence intervals, effect sizes, degrees of freedom and *P* value noted<br>*Give P values as exact values whenever suitable.*
☒	☐	For Bayesian analysis, information on the choice of priors and Markov chain Monte Carlo settings
☒	☐	For hierarchical and complex designs, identification of the appropriate level for tests and full reporting of outcomes
☐	☒	Estimates of effect sizes (e.g. Cohen's *d*, Pearson's *r*), indicating how they were calculated

Our web collection on statistics for biologists contains articles on many of the points above.

Software and code

Policy information about availability of computer code

Data collection	Data was collected by high-throughput DNA sequencing (Illumina MiSeq or NextSeq instruments). Standard Illumina software used to obtain sequences and quality scores.<br>CADD, phastCons, phyloP and GERP++ scores were downloaded from CADD v1.3 [http://cadd.gs.washington.edu/download]<br>FunSeq v2.16 scores were downloaded from http://archive.gersteinlab.org/funseq2.1.2/hg19_NCscore_funseq216.tsv.bg.<br>LINSIGHT scores downloaded from http://compgen.cshl.edu/~yihuang/tracks/LINSIGHT.bw<br>ReMM v0.3.1 scores were downloaded from https://charite.github.io/software-remm-score.html<br>GWAVA scores for the unknown, region and TSS models were calculated for all positions along the targeted regions using available software [ftp://ftp.sanger.ac.uk/pub/resources/software/gwava/v1.0/].<br>DeepSEA [http://deepsea.princeton.edu/job/analysis/create/] and fathmm-MKL [http://fathmm.biocompute.org.uk/fathmmMKL.htm] scores were retrieved using the respective online web interfaces.<br>DeltaSVM scores were computed from the precomputed k-mer weights [http://www.beerlab.org/deltasvm]. Using a custom Python script, for each variant the average of all possible k-mer scores of the alternative allele was subtracted from the average of all possible k-mer scores of the reference allele. Not available k-mers in the files are treated as zero.<br>JASPAR 2018 was downloaded from http://expdata.cmmt.ubc.ca/JASPAR/downloads/UCSC_tracks/2018/hg19/JASPAR2018_hg19_all_chr.bed.gz<br>TFBS predictions overlapping respective ChIP-peaks in ENCODE experiments were downloaded from http://compbio.mit.edu/encode-motifs/. TFBSs annotated in the Ensembl Regulatory Build v90 were downloaded from ftp://ftp.ensembl.org/pub/release-90/regulation/homo_sapiens and coordinate converted to GRCh37 using the UCSC liftover program.
Data analysis	For creating tag to sequence variant assignments, sequence reads were aligned using BWA-mem v0.7.10-r78972 with an increased penalty against local alignments (-L 80) to the Sanger determined references. A minimum coverage of three reads along the whole target was required to include variant calls from bcftools v1.273 for each identified tag.<br>For RNA/DNA count data, paired-end reads each sequenced the tags from the forward and reverse direction and allowed for adapter trimming and consensus calling of tags using previously described read merging (Kircher M, Methods Mol. Biol 2012). Tag or UMI reads containing unresolved bases (N) or those not matching the designed length were excluded using GNU command line tools. In the

downstream analysis using GNU command line tools, each tag x UMI pair is counted only once and only tags matching the above obtained assignment were considered.
RNA and DNA counts for each replicate were combined by tag sequence (using GNU command line tools), excluding tags not observed in both RNA and DNA of the same experimental replicate. All tags (T) not associated with insertions or multiple base-pair deletions were included in a matrix (converted using a custom Python script) of RNA count, DNA count, and N binary columns indicating whether a specific sequences variant was associated with the tag. We fit multiple linear regression models of the form log2(RNA) ~ log2(DNA) + N + offset using the R generalized linear model package and report the coefficients of N as effects for each variant. 95%-confidence intervals were calculated from the generalized linear model using the confint.lm method of the stats package in R.
Correlations, described statistical tests and Leave One Out Regression models were all performed in R (v3.1.0 and above).

For manuscripts utilizing custom algorithms or software that are central to the research but not yet described in published literature, software must be made available to editors/reviewers. We strongly encourage code deposition in a community repository (e.g. GitHub). See the Nature Research guidelines for submitting code & software for further information.

Data

Policy information about availability of data

All manuscripts must include a data availability statement. This statement should provide the following information, where applicable:

- Accession codes, unique identifiers, or web links for publicly available datasets
- A list of figures that have associated raw data
- A description of any restrictions on data availability

The sequencing data, obtained tag-to-variant assignments and processed RNA/DNA data have been submitted to the NCBI Gene Expression Omnibus (GEO; http://www.ncbi.nlm.nih.gov/geo/) under accession number GSE126550. The expression effect estimates and further information is available at DOI 10.17605/OSF.IO/75B2M. Plasmid construct sequences were deposited in NCBI GenBank (accessions MK484103.1 to MK484108.1).

Field-specific reporting

Please select the one below that is the best fit for your research. If you are not sure, read the appropriate sections before making your selection.

☒ Life sciences ☐ Behavioural & social sciences ☐ Ecological, evolutionary & environmental sciences

For a reference copy of the document with all sections, see nature.com/documents/nr-reporting-summary-flat.pdf

Life sciences study design

All studies must disclose on these points even when the disclosure is negative.

Sample size	MPRA was conducted using three technical replicates for each experiment. In addition, for LDLR and SORT1, independent MPRA libraries were created and cells were transfected from a different culture and on a different day (biological replicates). In one case (TERT), the same MPRA library was used for experiments in two different cell-types (HEK293T and a glioblastoma cell line).
Data exclusions	No data points were specifically excluded from the analyses. However, some analyses are restricted to a minimum of 10 tags per variant or a specified p-value threshold.
Replication	MPRA was conducted using three technical replicates for each experiment. In addition, for LDLR and SORT1 , independent MPRA libraries were created and cells were transfected from a different culture and on a different day (biological replicates). In one case (TERT), the same MPRA library was used for experiments in two different cell-types (HEK293T and a glioblastoma cell line).
Randomization	All MPRA data were allocated based on the regulatory element for which saturation mutagenesis was carried out.
Blinding	Blinding was not possible, as we needed to know the identity of each library for the subsequent analyses.

Reporting for specific materials, systems and methods

We require information from authors about some types of materials, experimental systems and methods used in many studies. Here, indicate whether each material, system or method listed is relevant to your study. If you are not sure if a list item applies to your research, read the appropriate section before selecting a response.

Materials & experimental systems

n/a	Involved in the study	
☒	☐	Antibodies
☐	☒	Eukaryotic cell lines
☒	☐	Palaeontology
☒	☐	Animals and other organisms
☒	☐	Human research participants
☒	☐	Clinical data

Methods

n/a	Involved in the study	
☒	☐	ChIP-seq
☒	☐	Flow cytometry
☒	☐	MRI-based neuroimaging

Eukaryotic cell lines

Policy information about cell lines

Cell line source(s)	All cell lines were obtained from ATCC and primary gliobastoma (GBM) cell line SF7996 49 was obtained from Dr. Joseph Costello's lab at UCSF.
Authentication	None of the cell lines were authenticated in our labs and used as provided by ATCC.
Mycoplasma contamination	We did not test for mycoplasma.
Commonly misidentified lines (See ICLAC register)	None
